# Emerging Transcriptional and Genomic Mechanisms Mediating Carbapenem and Polymyxin Resistance in *Enterobacteriaceae*: a Systematic Review of Current Reports

**DOI:** 10.1128/mSystems.00783-20

**Published:** 2020-12-15

**Authors:** Masego Mmatli, Nontombi Marylucy Mbelle, Nontuthuko E. Maningi, John Osei Sekyere

**Affiliations:** a Department of Medical Microbiology, Faculty of Health Sciences, University of Pretoria, Pretoria, South Africa; b Department of Microbiology, School of Life Sciences, University of KwaZulu-Natal, Durban, South Africa; University of Illinois at Chicago

**Keywords:** carbapenem resistance, polymyxin resistance, *Enterobacteriaceae*, transcription factors, resistance mechanisms, *Enterobacteriales*, antimicrobial resistance mechanisms, colistin, carbapenems

## Abstract

The spread of carbapenem- and polymyxin-resistant *Enterobacteriaceae* poses a significant threat to public health, challenging clinicians worldwide with limited therapeutic options. This review describes the current coding and noncoding genetic and transcriptional mechanisms mediating carbapenem and polymyxin resistance, respectively. A systematic review of all studies published in PubMed database between 2015 to October 2020 was performed. Journal articles evaluating carbapenem and polymyxin resistance mechanisms, respectively, were included. The search identified 171 journal articles for inclusion. Different New Delhi metallo-β-lactamase (NDM) carbapenemase variants had different transcriptional and affinity responses to different carbapenems. Mutations within the *Klebsiella pneumoniae* carbapenemase (KPC) mobile transposon, Tn*4401*, affect its promoter activity and expression levels, increasing carbapenem resistance. Insertion of IS*26* in *ardK* increased imipenemase expression 53-fold. *ompCF* porin downregulation (mediated by *envZ* and *ompR* mutations), *micCF* small RNA hyperexpression, efflux upregulation (mediated by *acrA*, *acrR*, *araC*, *marA*, *soxS*, *ramA*, etc.), and mutations in *acrAB*-*tolC* mediated clinical carbapenem resistance when coupled with β-lactamase activity in a species-specific manner but not when acting without β-lactamases. Mutations in *pmrAB*, *phoPQ*, *crrAB*, and *mgrB* affect phosphorylation of lipid A of the lipopolysaccharide through the *pmrHFIJKLM* (*arnBCDATEF* or *pbgP*) cluster, leading to polymyxin resistance; *mgrB* inactivation also affected capsule structure. Mobile and induced *mcr*, efflux hyperexpression and porin downregulation, and Ecr transmembrane protein also conferred polymyxin resistance and heteroresistance. Carbapenem and polymyxin resistance is thus mediated by a diverse range of genetic and transcriptional mechanisms that are easily activated in an inducing environment. The molecular understanding of these emerging mechanisms can aid in developing new therapeutics for multidrug-resistant *Enterobacteriaceae* isolates.

## Abstract (precis)

The spread of carbapenem- and polymyxin-resistant *Enterobacteriaceae* poses a significant threat to public health, challenging clinicians worldwide with limited therapeutic options. This review describes the current coding and noncoding genetic and transcriptional mechanisms mediating carbapenem and polymyxin resistance, respectively.

## INTRODUCTION

Antibiotic resistance is a significant problem worldwide, and its spread is a threat to public health and veterinary medicine due to the resultant restriction or depletion of therapeutic options, increased health care costs, unlimited transmission, and alarming mortality rates (1–3). Bacteria belonging to the *Enterobacteriaceae* family are of clinical concern owing to their association with carbapenem and polymyxin resistance worldwide (4). Specifically, carbapenem-resistant *Enterobacteriaceae* are designated by the World Health Organization (WHO) as critical priority pathogens due to their multidrug resistance (MDR) phenotypes and associated morbidities and mortalities (5). This is understandable since carbapenems are the treatment of choice and last-resort agents used against severe infections caused by MDR *Enterobacteriaceae*, which are usually resistant to clinically available antibiotics, including β-lactams, fluoroquinolones, and aminoglycosides (4, 6, 7). Although not categorized by the WHO yet, polymyxin-resistant Gram-negative bacteria (*Enterobacteriaceae*) also present a grave clinical challenge due to the importance of polymyxin as a reserved agent in treating carbapenem-resistant bacterial infections (8). The most frequently prescribed carbapenems include ertapenem, imipenem, and meropenem. Unlike other β-lactam antibiotics, carbapenems have the broadest antibacterial spectrum (2).

**FIG 1:**
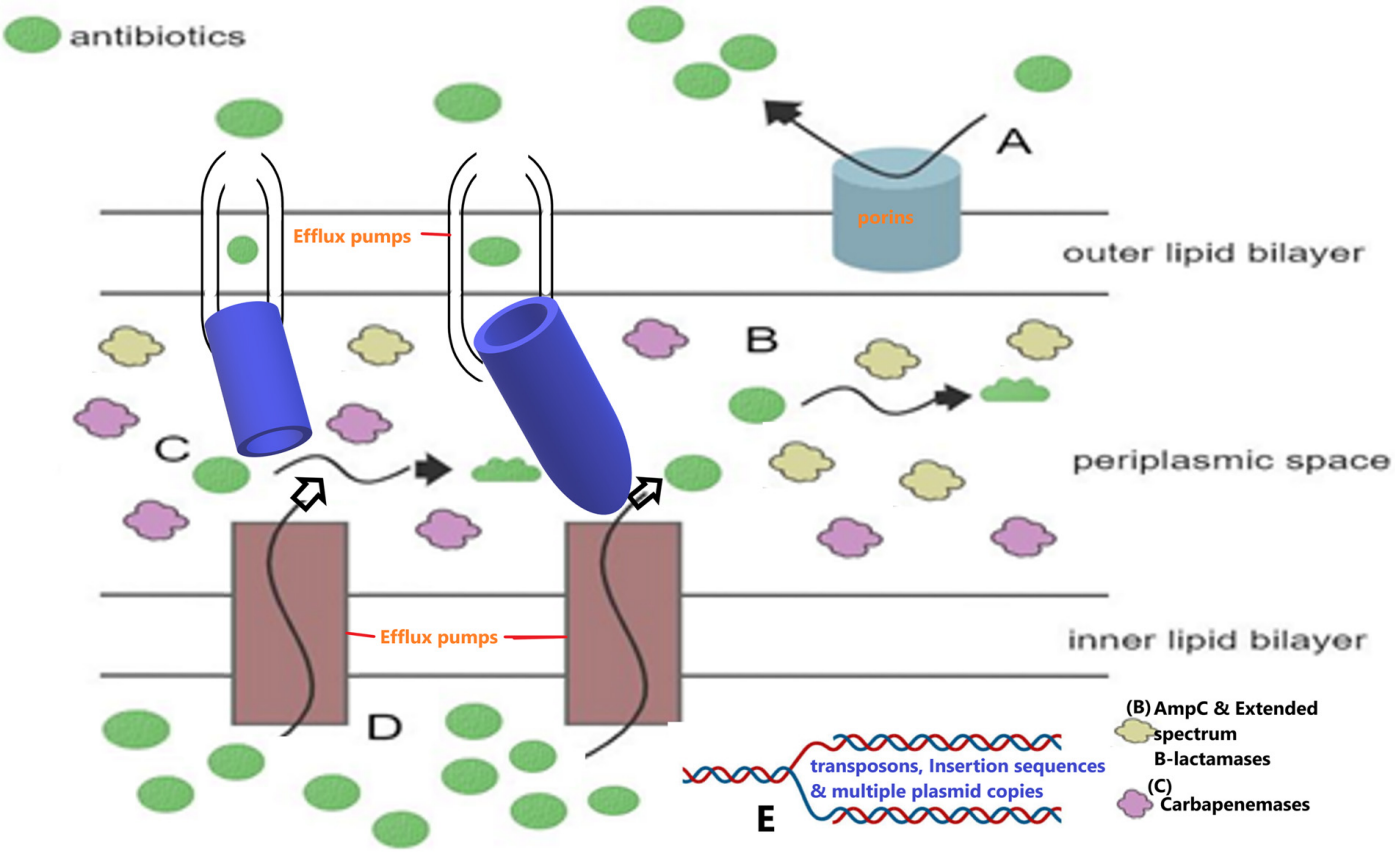
Summary of the carbapenem resistance mechanisms seen in Gram-negative bacteria (*Enterobacteriaceae*). (A) High-level resistance to carbapenems can be mediated by the alteration of membrane permeability due to porin mutations, restricting the entry of antibiotics into the periplasmic space. (B and C) Further, the hydrolysis of carbapenems (green balls) by highly expressed (increased concentrations of) AmpCs, and extended-spectrum β-lactamases (ESBLs) (B), as well as carbapenemases (C), respectively, also confer resistance to carbapenems. (D) Increased efflux pump activity also decreases the concentration of antibiotics (carbapenems) in the periplasmic space, reducing susceptibility to the antibiotic. (E) Finally, mobile genetic elements (MGEs), such as plasmids (with high copy numbers), transposons, and insertion sequences (upstream or within promoter sequences), can increase the expression levels of carbapenemases, AmpCs, and ESBLs, leading to higher levels of resistance to carbapenems. The figure was constructed using chemix.org and Paint 3D.

The use of carbapenems for MDR bacterial infections created a selection pressure within the clinical setting, resulting in the emergence of carbapenem resistance (2). Carbapenem resistance is primarily mediated by carbapenemase genes found on mobile genetic elements such as plasmids, integrons, insertion sequences, and transposons, allowing for easier horizontal transfer of genes across and within different bacterial species (9–12). Carbapenemases are a group of β-lactamases that hydrolyze the β-lactam ring of antibiotics, rendering them inactive (11). Other carbapenem resistance mechanisms include porin alteration, target modification, overproduction of extended-spectrum β-lactamases (ESBLs), and overexpression of efflux pumps (Fig. 1) (13).

The emergence of carbapenem resistance in *Enterobacteriaceae* led to the reintroduction of polymyxin as a therapeutic option (14). Polymyxins, which are made up of polymyxin E and B (also known as colistin), were discovered in 1947 and used for the treatment of Gram-negative bacterial infections. Polymyxin acts by binding to the lipid A of the outer membrane of Gram-negative bacteria (15, 16). This interaction results in the disruption of the bacterial membrane, leading to cell death (13, 15). The use of polymyxin, however, was diminished in the 1970s due to its neurotoxicity and nephrotoxicity (16) but was widely used in veterinary medicine for the treatment of diarrhea in food-producing animals and as a growth promoter (17, 18). Its use as a growth promoter resulted in increased reports on polymyxin-resistant bacteria. Thereafter, a worldwide dissemination of the plasmid-mediated *mcr-1* gene through the food-chain proliferated (19). Hence, several countries banned or restricted the use of polymyxin as a feed additive in veterinary medicine (20). Despite polymyxin’s limitations in human medicine, the increasing incidence of carbapenem-resistant *Enterobacteriaceae* led to the revival of polymyxin as a last-line treatment (21, 22).

The use of polymyxin in both human and veterinary medicine has led to the emergence of polymyxin-resistant *Enterobacteriaceae* (8). Polymyxin resistance is primarily mediated through covalent modification of the lipid A moiety of the bacterial lipopolysaccharide (LPS), through the addition of 4-amino-4-deoxy-arabinose or phosphoethanolamine residues (8, 23, 24). Specific chromosomal mutations within the two-component systems *pmrA/pmrB* and *phoP/phoQ* cause these modifications and within the genes that regulate these systems (23, 25). Recently, plasmid-mediated *mcr*-type genes encoding a phosphoethanolamine transferase enzyme were discovered and found to be responsible for the horizontal transfer of polymyxin resistance (8, 25). These modifications reduce the negative net charge of the LPS, reducing the affinity of the polycationic polymyxin peptide to the outer membrane of bacteria and thus decreasing the bacterial susceptibility to polymyxin (8, 15). Other resistance mechanisms include the use of efflux pumps, the formation of capsules, and the decrease in outer membrane proteins (8).

The occurrence of carbapenem and polymyxin resistance within *Enterobacteriaceae* reduces the therapeutic options for the treatment of MDR bacterial infections and increases the incidence of infections and mortality rates. This systematic review aims to describe the current resistance mechanisms that are known and explained in literature and to identify gaps within the field.

## EVIDENCE BEFORE THIS REVIEW

In terms of polymyxin resistance mechanisms, numerous reviews have evaluated both the different resistance mechanisms and the epidemiology of *mcr*-type genes and their role in mediating polymyxin resistance (26, 27). Carbapenem resistance mechanisms have also been described in several reviews for both carbapenemase-producing and -nonproducing *Enterobacteriaceae* (28). This review, however, provides an in-depth characterization of emerging coding and noncoding genomic and transcriptional mechanisms that mediate resistance in polymyxin- and carbapenem-resistant *Enterobacteriaceae*. Particularly, other genome-based but noncoding elements mediating polymyxin and carbapenem resistance are also highlighted since their roles in polymyxin and carbapenem resistance have been less reviewed. Each resistance mechanism is supplemented with evidence from the literature to emphasize its role in mediating resistance.

## LITERATURE SEARCH STRATEGY

A comprehensive literature search was carried out using the PubMed database. English journal articles published within the last 5 years (January 2015 to October 2020) were retrieved and screened with the following keywords: “carbapenem” and “colistin” in permutation and combination with “imipenem OR ertapenem OR meropenem OR doripenem OR polymyxin” and “resistance AND *Enterobacteriaceae*” in a factorial fashion. The search was focused on journal articles that evaluated the role of specific genes, noncoding elements, and transcriptional factors mediating resistance in *Enterobacteriaceae* using molecular intervention (mutagenesis, gene editing, etc.) assays. Therefore, studies that involved reviews, diagnostics, case reports, case studies, risk factors, epidemiology, and surveillance were excluded. Studies that performed antibiotic sensitivity testing and identified the presence of carbapenemase and *mcr-*type genes in isolates without evaluating the role of genes in mediating resistances were regarded as epidemiological studies and were excluded. Nevertheless, epidemiological studies that identified these resistance genes and further investigated whether the transfer of genes conferred resistance were included. The following data were extracted from the included articles: *Enterobacteriaceae* species, sample sources, molecular techniques used, and resistance genes (Table 1). The inclusion and exclusion protocols used in this review are given in Fig. S1 in the supplemental material.

**TABLE 1:** Emerging genomic and transcriptional mechanisms mediating carbapenem resistance mechanisms in *Enterobacteriaceae*

Resistance mechanism	Species	Resistance determinant(s)	Reference(s)
Narrow- and extended-spectrum β-lactamases	Enterobacter cloacae complex	AmpC	100
	Escherichia coli	AmpC	101
		ESBL	1, 54, 58
	Klebsiella pneumoniae	AmpC, ESBL	101
Carbapenemase	Citrobacter freundii	LMB-1	39
		NDM, KPC	11
		OXA	102, 103
	Escherichia coli	IMP	47
		KPC-variants	3, 37, 46
		NDM	3, 44, 45, 51, 103–109
		NDM, KPC, IMP	110
		OXA	41, 111, 112
	Enterobacter cloacae complex	FRI-3	113, 114
		FLC-1	115
		GES	116
		IMP	115
		KPC	46
		LMB	117
		MIR-17	118
		NDM	119
		OXA	102, 120
	Klebsiella pneumoniae	KPC	9, 32, 121–126
		IMP	127
		NDM-4	43, 44, 128–132
		OXA	42, 102, 111, 130, 133–136
		VIM	125, 137
	Klebsiella aerogenes	NDM	138
	Klebsiella quasipneumoniae	KHM-1	139
	E. coli, E. cloacae, and K. pneumoniae	NDM	12
Efflux pumps	Escherichia coli	AcrAB-TolC	3, 55, 59, 63, 65, 67, 140
	Klebsiella pneumoniae	AcrAB-TolC	3, 65, 67, 125
		AcrAB, RamA	141
Porin deficiency	Escherichia coli	OmpK35 and/or OmpK36	49, 51, 52, 54, 58, 67, 101, 125, 142, 143
		PhoE	144
	Enterobacter cloacae complex	OmpK35 and/or OmpK36	1, 9, 64, 100, 145
		MicC and MicF	53
	Klebsiella pneumoniae	OmpK35 and/or OmpK36	32, 33, 67, 111, 125, 136, 145–147
	*Raoultella orithinolytica*	OmpK35 and/or OmpK36	6
Target modifications	Escherichia coli	MdrA	148, 149

10.1128/mSystems.00783-20.1 FIG S1 Flow chart showing the literature search strategy, inclusion and exclusion criteria, and the final number of manuscripts used for the review. Download FIG S1, PDF file, 0.1MB. Copyright © 2020 Mmatli et al. 2020 Mmatli et al. This content is distributed under the terms of the Creative Commons Attribution 4.0 International license.

## CARBAPENEM RESISTANCE MECHANISMS

Carbapenem resistance in carbapenem-resistant *Enterobacteriaceae* (CRE) is mediated by a variation and synchrony of different resistance mechanisms: the loss of major porin proteins, increased activity of efflux pumps, and the production of β-lactamases, i.e., carbapenemases, ESBLs, and cephalosporinases (AmpCs) (Fig. 1). The β-lactamases include ESBLs (TEM, PER, VEB, SHV, LEN, and CTX-M), carbapenemases (such as KPC, GES-5, IMI, VCC, OXA-48, IMP, VIM, and NDM), and AmpC-type β-lactamases (i.e., CMY, FOX, MOX, ACT, MIR, DHA, etc.) (29–31). These enzymes are frequently plasmid-borne genes, allowing for the dissemination of genes within and between Gram-negative bacteria species. This results in resistance toward penicillin, cephalosporins, carbapenems, and/or monobactams (29, 202, 203). Carbapenem resistance in *Enterobacteriaceae* is mediated mainly by the production of carbapenemases (11). However, elevated carbapenem resistance in carbapenemase-producing *Enterobacteriaceae* is usually through the overproduction of carbapenemases and/or alteration of membrane permeability (Table 1) (32).

## CARBAPENEMASE PRODUCTION

Carbapenemases are broad-spectrum β-lactamases that hydrolyze the β-lactam ring of carbapenems and other β-lactam antibiotics (11, 33). There are three groups of carbapenemases: Ambler class A, class B that is made up of metallo-β-lactamases (MBLs), and class D β-lactamases (34–36). Class A has a broad spectrum of activity and utilizes a serine residue in its active site during cleavage of the β-lactam ring of penicillin, cephalosporins, classic β-lactamase inhibitors (sulbactam and tazobactam), aztreonam, and carbapenems (37, 38). Class B depends on zinc as a cofactor in its active site but has a similar spectrum of activity as class A, sparing aztreonam (39, 40). Class D, similar to class A, utilizes a serine residue in its active site but has a unique spectrum of activity, i.e., reduced carbapenem susceptibility, high resistance to penicillin, and intermediate resistance to cephalosporins; it is inactive against aztreonam (41, 42). Klebsiella pneumoniae carbapenemase (KPC) of class A, New Delhi MBL (NDM) carbapenemase of class B, and oxacillin-hydrolyzing carbapenemase (OXA-48/-181) from class D are responsible for most carbapenem resistance in CRE (35).

Other clinically relevant carbapenemases include MBLs that belong to subclass B1: Verona Integron-encoded MBLs (VIM) and imipenemase (IMP) (34, 43). As carbapenemases spread within *Enterobacteriaceae*, amino acid substitutions occur, producing different variants of the carbapenemase. This results in changes in the carbapenemase activity and its affinity to carbapenems (43). Paul et al. (44) showed that NDM variants (NDM-1 and NDM-5) had different transcriptional responses to different carbapenems, where NDM-5 had a 10-fold increase in expression when exposed to ertapenem, and NDM-1 had only a 2-fold increase. Paul et al. (44) further speculated that new variants of NDM are evolving inducibility in the presence of carbapenem drugs, resulting in elevated NDM production. A similar study revealed how molecular differences between NDM-17 and NDM-5 carbapenemases affected their carbapenemase activity (45). NDM-17 had an E170K (glutamic acid to lysine) amino acid substitution that was responsible for higher affinity and an increased carbapenemase activity compared to NDM-5, resulting in elevated ertapenem and meropenem resistance (45). In KPC, mutations within its mobile transposon affects the promoter activity of *bla*_KPC_ and, subsequently, carbapenem resistance (Table 1).

The *bla*_KPC_ gene is usually located within a 10-kb mobile transposon, Tn*4401*, allowing for its dissemination within the *Enterobacteriaceae* family and other Gram-negative bacteria such as *Pseudomonas* and Acinetobacter species (46). Cheruvanky et al. studied the different Tn*4401* isoforms within *Enterobacteriaceae* and identified three isoforms—Tn*4401b*, Tn*4401a*, and Tn*4401h*—which were mostly found in *Klebsiella* (48%), *Enterobacter* (37%), and *Citrobacter* (12%) spp. Tn*4401a* and Tn*4401h* were mutational variations of Tn*4401b*. Genomic comparison analysis found that Tn*4401a* and Tn*4401h* had 99- and 188-bp deletions, respectively, between the P1 and P2 regions of the putative promoter sequences (46). These mutations increased the promoter activity of these isoforms that resulted in a 23- and 4-fold increase in KPC expression in Tn*4401a* and Tn*4401h*, respectively, compared to the Tn*4401b* isoform (46).

In electrocompetent E. coli Genehog cells, the three different isoforms were introduced. This resulted in meropenem MIC values of 1, 16, and 4 μg/ml for Tn*4401b*, Tn*4401a*, and Tn*4401h*, respectively. Tn*4401a* had the highest KPC production, which conferred the highest meropenem resistance (46). Huang et al. performed a similar study characterizing three Tn*3*-Tn*4401* chimera isoforms: CTA, CTB, and CTC. The chimeras had different combinations of P1, PY, and PX promoters, and the study evaluated how it affected the expression of *bla*_KPC_ and carbapenem susceptibility in KPC-producing isolates. Huang et al. and Cheruvanky et al. both showed that mutations within the putative promoter sequence of Tn*4401* affect the expression of the *bla*_KPC_ gene and carbapenem susceptibility in isolates.

The overproduction of carbapenemase in an IMP-harboring E. coli isolates was achieved through the insertion of an insertion element (IS*26*) within the *ardK* gene of the IncN plasmid during meropenem selection (47). *ardK* encodes a putative transcription factor that negatively modulates the transcription of the *bla*_IMP-6_ gene (47). The disruption of this gene with an IS*26* element resulted in 53- and 256-fold increases in IMP production and meropenem resistance, respectively (47). Although the parental E. coli strain harbored IMP and was carbapenem susceptible, it plays a potential role in the dissemination of IMP-6-harboring plasmids that can, under selection, mediate a high-level carbapenem resistance (47). Wu et al. (11) identified a clinical Citrobacter freundii ST88 isolate that harbored two carbapenemase-harboring plasmids encoding *bla*_KPC-2_ and *bla*_NDM-1_, respectively. The transformation of E. coli J53 with both plasmids conferred 2- or 4-fold increases in imipenem and meropenem MICs compared to J53 isolate alone or with either plasmid. Thus, the coexistence of *bla*_KPC-2_ and *bla*_NDM-1_ resulted in a synergistic effect, conferring high-level carbapenem resistance, resulting in MIC values of 1,024 and 512 μg/ml for imipenem and meropenem, respectively, in the C. freundii isolate (Table 1).

Unlike the other resistance mechanisms, carbapenemase production is adequate to confer clinical resistance for carbapenems. This was shown by Choudhury et al., who transformed E. coli J53 competent cells with an NDM-4-harboring plasmid. The donor isolate, E. coli ST448, and the transformant both had MICs above the imipenem, meropenem, and ertapenem breakpoints (48).

## β-LACTAMASE PRODUCTION

In non-carbapenemase-producing carbapenem-resistant *Enterobacteriaceae*, carbapenem nonsusceptibility is observed in ESBL- and/or AmpC β-lactamase-producing *Enterobacteriaceae*. When ESBL production is coupled with the loss of the two major outer membrane porin groups, including OmpC and OmpF, clinical carbapenem resistance is observed (49). OmpC and OmpF are responsible for the nonspecific transport of solutes across the outer membrane into the cytoplasm (4). OmpC and OmpF are homologues of OmpK36 and OmpK35, respectively (6), and will be used interchangeably in this review.

The production of extended-spectrum and AmpC β-lactamases alone without membrane impermeability is insufficient to confer clinical carbapenem resistance. van Boxtel et al. (1) showed that the transformation of E. coli isolates with a plasmid encoding *bla*_CMY-2_ gene resulted in reduced meropenem susceptibility of E. coli but did not confer clinical meropenem resistance (Table 1).

AmpC and ESBLs have previously been shown to hydrolyze carbapenems weakly, and van Boxtel et al. further showed that CMY-2 hydrolysis of meropenem was below the detection limit during β-lactamase activity evaluation assays. Clinical meropenem resistance was observed when a *bla*_CMY-2_- harboring plasmid transformed a porin-deficient Escherichia coli isolate (1). Guiana ESBL (GES) belonging to Amber class A acquires carbapenemase activity due to the glycerin substitution at position 170 with either a serine or an asparagine (13). Streling et al. (13) reported that GES-16, which had the Gly170Ser amino acid substitution, had a broad-spectrum hydrolysis profile, hydrolyzing penicillin, cephamycin, cephalosporins, and carbapenems. Although GES-16 showed some carbapenemase activity, it conferred low-level resistance to carbapenems. Moreover, it remains to be seen if GES-16 shall be classified as a carbapenemase like GES-5 (50) since they had similar kinetic parameters toward carbapenems (13).

## ALTERATION OF MEMBRANE PERMEABILITY

The loss of major outer membrane proteins, OmpK36 and OmpK35, is frequently observed in CRE, resulting in reduced permeability of the outer membrane due to structural changes in porin channels restricting the uptake of charged molecules through the bacterial cell wall (Fig. 1) (3, 49, 51). These structural changes were due to mutations that either reduced the channel size of porins or modified its electrostatics (52). This phenotype is frequently observed in resistance-induced mutants through serial passage assays of non-carbapenemase-producing isolates (53). Hao et al. (53) revealed that clinical *Enterobacteriaceae* isolates acquired carbapenem resistance through mutations within both OmpK36 and OmpK35 proteins. Isolates with mutations in both OmpK36 and OmpK35 had elevated resistance (8 to 32 μg/ml) to ertapenem, imipenem, and meropenem compared to isolates with only OmpK36 mutations (0.25 to 2 μg/ml). This correlates with the findings of Hamzaoui et al. (2) in K. pneumoniae isolates, which showed that the loss of both major porins or mutations within genes seen to regulate the porin system resulted in elevated carbapenem resistance. These genes include the two-component transduction regulatory system *envZ-ompR*, *micF*, and *micC* genes (4, 53). These mutations include single-base deletion, insertion, or substitution in the coding sequence resulting in the inactivation of proteins (53).

EnvZ is an inner membrane sensor kinase that is encoded by the EnvZ-OmpR regulatory system that regulates the expression of the two major porin groups, OmpC and OmpF (54). This is accomplished through phosphorylation or dephosphorylation of OmpR, the transcriptional factor responsible for porin gene activation (54). The phosphorylation of OmpR results in a structural change in the protein, increasing its binding affinity to the major porin transcriptional factor binding site (51). Kong et al. (51) revealed that the Gly63Ser amino acid substitution within the N-terminal phosphorylation domain of OmpR affects the phosphorylation of OmpR by EnvZ. The OmpR mutant, after that, failed to initiate porin transcription, resulting in a change in membrane permeability and, subsequently, carbapenem resistance (51). The study further went on to show the synergistic effect of OmpR mutants and carbapenemase activity, with the transformation of OmpR mutants with a NDM-harboring plasmid resulting in a 100-fold increase in the carbapenem MIC (Table 1) (51).

SDS-PAGE analysis of the outer membrane porins of OmpR mutants revealed a sharp decrease in the expression of both major porin groups (49). Adler et al. (55) reported that mutations within both *envZ* and *ompR* are early genetic events that mediate carbapenem resistance during serial passage. OmpR mutants had decreased expression of both *ompC* and *ompF*, whereas *envZ* mutations led to downregulation of *ompF* and upregulation of *ompC*. In *ompCF*-deleted porin-deficient isolates, *envZ* mutations were still observed and resulted in a 6-fold increase in carbapenem resistance, illustrating that *envZ* mutations are critical for carbapenem resistance (55). The role of *envZ* in mediating resistance in porin-deficient isolates is still unknown (54).

The loss of the major porin groups can also be achieved through the change in expression of *micC* and *micF* genes. These genes are part of the outer membrane genes and encode small antisense RNA that negatively regulates OmpC and OmpF genes (4, 53). Serial passage of Enterobacter aerogenes (currently Klebsiella aerogenes) performed by Hao et al. (53) produced two carbapenem-resistant isolates with the loss of both major porin groups. SDS-PAGE analysis of the outer membrane proteins revealed the loss of both porins; however, no mutations within *ompCF* were observed compared to *E. aerogenes* strain NCTC10336, which led to the investigation of *micC* and *micF* gene expression. Transcriptional analysis revealed that the overexpression of both *micC* and *micF* in isolates results in the significant downregulation of OmpK36 and OmpK35, respectively (53). Similar results were observed in E. coli, where the upregulation of *micF* resulted in the downregulation of *ompF* genes (4). Interestingly, the upregulation of *micC* leads to the downregulation of *ompC* but to an increase in *ompF* to compensate for the loss of OmpC (4).

## PORIN DEFICIENCY AND β-LACTAMASE PRODUCTION

The loss of both major outer membrane proteins, OmpK35 and OmpK36, in E. coli and K. aerogenes was seen in some clinical isolates to mediate high-level carbapenem resistance (51–53). However, in K. pneumoniae, Salmonella enterica serotype Typhimurium, and other *Enterobacteriaceae* species, including Enterobacter cloacae complex (E. asburiae and E. cloacae) and Raoultella ornithinolytica, porin deficiency reduces the susceptibility of isolates to carbapenem but does not confer clinical resistance, and β-lactamase activity is required (56, 57). The transformation of porin-deficient isolates with an NDM-harboring plasmid revealed a synergistic effect mediating high-level carbapenem resistance (51). Porin deficiency reduces the uptake of antibiotics into the periplasm, which reduces the concentrations of antibiotics in the periplasmic space and cytosol, amplifying the β-lactamase effect (due to reduced intracellular antibiotic concentrations) and resulting in a synergistic effect (Fig. 1) (49). van Boxtel et al. (1) demonstrated that for clinical meropenem resistance in CMY-2-harboring E. coli isolate, mutations that disrupt porin expression and increase CMY-2 expression were required. Individually, the loss of porin and the upregulation of CMY-2 expression reduced meropenem susceptibility in isolate but did not confer clinical resistance. Clinical resistance was achieved when both mechanisms were found in the isolate, resulting in a meropenem MIC of >32 μg/ml (1) (Table 1 and Fig. 1).

## OVERPRODUCTION OF EFFLUX PUMPS

In ESBL-producing *Enterobacteriaceae*, carbapenem resistance is also achieved through the overexpression of efflux pumps (58, 59). The overexpression of the efflux pump phenotype is usually observed when there is a significant increase in carbapenem susceptibility when an isolate is incubated with a carbapenem and the appropriate efflux pump inhibitor. There are different types of efflux pump inhibitors (EPIs) based on their mechanisms of action (60). Carbonyl cyanide *m*-chlorophenylhydrazine (CCCP) is an example of a protonophore that indirectly affects the activity of proton pumps by disrupting the proton motive force, reducing ATP production and resulting in an increased membrane permeability (60, 61). The disruption of the proton motive force across the membrane leads to membrane depolarization, eradicating the electrochemical concentration gradient across the membrane (60).

Osei Sekyere and Amoako (60) hypothesized that the cytoplasmic ion imbalance caused by the depolarized membrane created by CCCP disrupts the optimal activity of carbapenemase, which requires energy (ATP) and zinc to function. This was observed when some carbapenemase-producing *Enterobacteriaceae* species resulted in a 2-fold reduction in meropenem resistance in the presence of CCCP. At the same time, CCCP did not affect carbapenem susceptibility in non-carbapenemase-producing *Enterobacteriaceae* isolates (60). More research evaluating the effects of a depolarized membrane on carbapenemase activity is required to support this hypothesis.

Another mechanism of action of EPIs is the direct binding of an EPI to the functional efflux pump, reducing the ability of antibiotics to be pumped out of the cell by efflux pumps. This is the mechanism of action of phenylalanine-arginine β-naphthylamide (PAβN) (61). Lee and coworkers identified an E. cloacae ST74 clinical isolate whose imipenem susceptibility increased from 64 mg/ml to 0.5 mg/ml in the presence of PAβN, revealing the active role of efflux pumps in mediating carbapenem resistance and the synergistic effect of PAβN and carbapenemases in increasing carbapenem susceptibility in E. coli (Fig. 1) (60, 62).

AcrAB-TolC is a well-known multidrug efflux pump system that confers resistances toward a wide variety of agents, including β-lactams and is responsible for the MDR phenotype in E. coli (59, 63). It belongs to the resistance nodulation division (RND) superfamily and has been shown to synergistically work with other mechanisms to confer high-level resistance (3, 59). AcrAB-TolC is a tripartite efflux pump system that is made up of *acrA*, *acrB*, and *tolC* genes that encode a periplasmic membrane fusion protein, an inner membrane transporter, and an outer membrane protein, respectively (Fig. 1) (3, 59).

Saw et al. (3) evaluated the role of the AcrAB-TolC efflux pump system in *Enterobacteriaceae* species, *viz.*, E. coli, K. pneumoniae, and Salmonella enterica serotype Typhimurium. In all three isolates, mutations within *acrAB* and *tolC* had no significant effect on carbapenem susceptibility. The transformation of *acrAB* and *tol*C mutants of E. coli and K. pneumoniae with KPC-harboring plasmid resulted in 4- and 8-fold increases in ertapenem and meropenem resistance, respectively. In KPC-producing E. coli and K. pneumoniae, the introduction of *acrAB* mutations resulted in a 4-fold increase in ertapenem MIC in E. coli and a 2- to 8-fold increase in carbapenem resistance in K. pneumoniae. *acrAB* mutations in carbapenemase-harboring K. pneumoniae and E. coli isolates created a synergistic effect with the β-lactamase activity, causing high-level carbapenem resistance (3).

The introduction of *tolC* mutations and KPC- and NDM-harboring plasmids into *S.* Typhimurium isolates resulted in 2-, 250-, and 1,000-fold increases in the ertapenem MICs, respectively. The introduction of carbapenemases in Salmonella enterica serotype Typhimurium isolates, therefore, results in elevated carbapenem resistance (3). The introduction of *acrAB* mutations in KPC and NDM-producing isolates did not affect nor contribute to carbapenem resistance in *S.* Typhimurium isolates. In comparison, the introduction of *tolC* mutations resulted in 2- and 4-fold increases in the ertapenem MICs in *S.* Typhimurium isolates (3).

AcrAB-TolC efflux pump systems in the *Enterobacteriaceae* family are regulated by the local regulators AcrR and the global regulators MarA, SoxS, and RamA (59, 64). Mutations within *ramA* and *ramB* have been shown via quantitative-PCR to upregulate RamA and AcrA transcripts, increasing efflux pump activity and decreasing *ompCF* expression (64). A novel AraC-type regulator called regulator of antibiotic resistance A, RarA, regulates the efflux pump system conferring the MDR phenotype in *Enterobacteriaceae* (63). Chetri et al. (63) evaluated its transcriptional response with the increase of carbapenem concentration. The expression of RarA was directly proportional to the concentration of ertapenem, resulting in the upregulation of AcrAB expression, reducing carbapenem susceptibility in E. coli clinical isolates (Table 1).

The study showed that RarA acts as a positive regulator of AcrAB, independent of the global regulators MarA, SoxS, and RamA (63). The transformation of E. coli DH5α with a plasmid encoding *rarA* resulted in MIC values of >32 μg/ml for ertapenem, meropenem, and imipenem (65). Pavez et al. evaluated the AcrAB efflux pump expression under imipenem stress and found that MarA and SdeR were responsible for the increased expression of AcrAB efflux pump in E. coli, E. cloacae, and K. pneumoniae (65). The global regulatory pathways are interconnected and function to downregulate porin expression and upregulate efflux systems (64). The overexpression of *acrAB* decrease the expression of porin genes, *ompC* and *ompF* (59). Pal et al. (66) reported an active role of *acrB* expression in augmenting *ompC* reduction in E. coli and K. pneumoniae isolates. The mechanisms mediating this role is unknown; however, the global regulatory system is assumed to play a role (64). Mutations within AcrD, a transporter of the RND superfamily, have been shown to compensate for the loss of AcrB, increasing the export of carbapenems out of the periplasm and mediating carbapenem resistance (Fig. 1) (55). This was seen in E. coli isolates with *acrD* and *acrB* double mutants (55). Mutations in the local AcrAB-TolC regulator, AcrR, increases AcrB expression, increasing carbapenem nonsusceptibility in E. coli isolates (55). An increase in efflux pump activity, mediated by mutations in the regulators stated above, only confers clinical carbapenem resistance in *Enterobacteriaceae*, and aids in mediating high-level carbapenem resistance when coupled with β-lactamase/carbapenemase production (Fig. 1) (3, 67).

## POLYMYXIN RESISTANCE MECHANISMS

**FIG 2:**
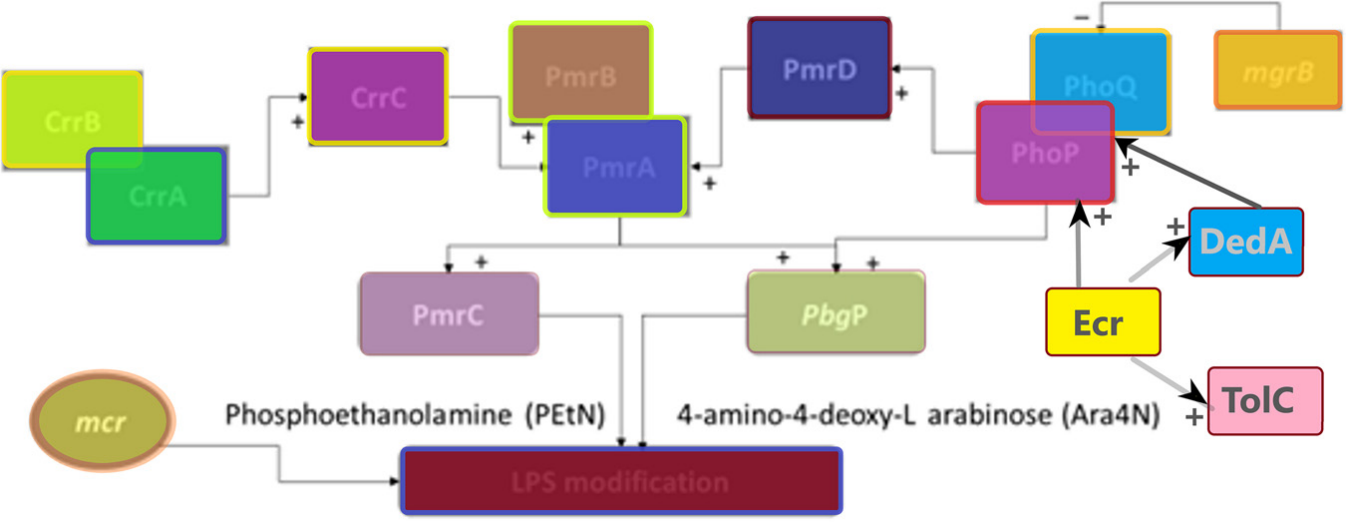
Representation of the various mechanisms and determinants interacting to mediate polymyxin resistance in Gram-negative bacteria. The LPS of the outer membrane of Gram-negative bacteria is modified through PmrC, the *pbgP* operon, and *mcr*-type genes. The PmrC and PbgP genes are regulated by three two-component systems—PhoPQ, PmrAB, and CrrAB—that are interconnected by CrrC and PmrD proteins. The newly discovered DedA and Ecr proteins also activate PbgP through PhoPQ, whereas Ecr also activates DedA and TolC. Heteroresistance in *Enterobacter* sp. is thought to be mediated by Ecr membrane proteins. A “+” indicates activation toward upregulation; a “–” indicates repression/inhibition toward downregulation. The diagram was constructed using Paint 3D with a structure based on one by Cheng et al. (75).

Polymyxin resistance in *Enterobacteriaceae* includes modification of the LPS in the outer membrane layer of the bacterium, neutralizing the negative charge of the outer membrane (23). This results in a weak interaction or binding affinity between the positively charged polymyxin and LPS molecules, *viz.*, lipid A (21, 22). These modifications include the transfer of 4-amino-4-deoxy-l-arabinose (Ara4N) and phosphoethanolamine (PEtN) to the 4-phosphate and the 1-phosphate groups of lipid A, respectively, through the *pbgP* operon and PmrC or *mcr*-type gene products (Fig. 2) (22).

The *pbgP* operon encodes the endogenous LPS modification system that is regulated by PhoPQ and PmrAB two-component regulatory systems (68, 69). The *pbgP* operon encodes enzymes that synthesize Ara4N from UDP glucuronic acid and mediates the addition of Ara4N to the 1-phosphate group of lipid A (70). These regulatory systems are responsible for the biosynthesis and transfer of Ara4N to lipid A; chromosomal mutations within the *phoP*, *phoQ*, and *pmrB* genes upregulate these systems, mediating polymyxin resistance (Fig. 2) (23, 71).

## PmrAB TWO-COMPONENT REGULATORY SYSTEM

The PmrAB system is made up of a *pmrABC* operon that encodes three proteins: a cytoplasmic membrane-bound sensor kinase, PmrB; a regulatory protein, PmrA; and a PEtN transferase, PmrC (21). PmrB activates PmrA protein through phosphorylation, which then binds to the *pbgP* operon for Ara4N modification (21). The cytoplasmic membrane-bound kinase is further activated by extracellular stimulants, such as a high concentration of iron (Fe^3+^) and aluminum (Al^3+^), as well as an acidic pH (<5.5) (69). Mutations within *pmrB* increase PmrB kinase activity, resulting in autophosphorylation of PmrA and leading to an increased expression of the *pbgP* operon (21). Cannatelli et al. (204) showed that *pmrB* mutation leads to the constitutive activation of *pmrA* that increases the expression of *pmrK* (of *pbgP* operon), resulting in colistin resistance in the E. coli ST59 isolate.

In Salmonella enterica subsp. *enterica* serovar Newport ST45, colistin resistance was achieved through a 12-nucleotide deletion within *pmrB*, conferring colistin resistance (16 mg/liter) (72). Phan et al. (21) showed that *pmrB* mutations have a feedback loop onto *pmrC* and *pmrA* genes located upstream of the *pmrB* gene. PmrC (also known as *eptA*) is responsible for the biosynthesis of PEtN transferase that is regulated by the response regulator PmrA (21). Hence, the upregulation of PmrA by PmrB mutants activates both *pmrC* and *pbgP*, resulting in both PEtN and Ara4N modification of the LPS (Fig. 2) (16, 73). The deletion of *pmrAB* decreases the expression of *pmrC* and *pbgP* operon and colistin susceptibility (Table 2) (22).

**TABLE 2:** Emerging genomic and transcriptional mechanisms mediating polymyxin resistance mechanisms in *Enterobacteriaceae*

Resistance mechanism	Species	Resistance determinant(s)	Reference(s)
Efflux pumps	Escherichia coli	AcrAB	150
		MarA, AcrAB	151
	Enterobacter cloacae complex	TolC, SoxRS	152
	Klebsiella pneumoniae	RamA, SoxS	80
	Salmonella enterica serovar Typhimurium	AcrB, CpxR	153
*mcr*-type genes	Citrobacter braakii	MCR-1	154
	Citrobacter freundii	MCR-1	155
	Escherichia coli	MCR-1	19, 82, 85, 93, 155–175
		MCR-2	176
		MCR-3	162, 169, 177, 178
		MCR-5	95, 96
		MCR-9	98
	*Enterobacteria cloacae* complex	MCR-4.3	97
	Klebsiella pneumoniae	MCR-1	25, 174, 179
		MCR-7.1	94
		MCR-8	180
	Salmonella enterica	MCR-1	181
		MCR-2	181
		MCR-9	92
	Salmonella enterica serovar Paratyphi B	MCR-5	99
	Salmonella enterica serovar Typhimurium	MCR-9	91
	Shigella sonnei	MCR-1	182
	Shigella flexneri	MCR-1	183
TCS PEtN modification	*Citrobacter*	PhoPQ-PmrAB	184
	Escherichia coli	PhoPQ-PmrAB	18, 21, 23, 24, 68, 185
		QseBC-PmrAB	98, 186, 187
	*Enterobacter*	PhoPQ-PmrAB	76, 184
	Klebsiella pneumoniae	PhoPQ-PmrAB	69, 71, 81, 188–190
		CrrAB	16, 22, 73–75, 77
	Salmonella enterica serovar Typhimurium	PhoPQ-PmrAB	72, 191
	Yersinia pestis	PhoPQ	192
PEtN modification	Escherichia coli	MgrB	68, 82, 83, 86
	Klebsiella pneumoniae	MgrB	7, 25, 133, 193–197
	Escherichia coli, Klebsiella pneumoniae, and Salmonella enterica	EptA	181, 185
	Escherichia coli	EptA, EptB, EptC	198
	Salmonella enterica serovar Typhimurium	MgrB, SroC, EptB	199
Membrane permeability	Salmonella enterica serovar Typhimurium	Mig-14	200
Unknown mechanisms	Salmonella enteritidis	Not applicable	201

## CrrAB TWO-COMPONENT REGULATORY SYSTEM

The PmrAB regulatory system is regulated by the CrrAB two-component regulatory system, which encodes a sensor kinase (*crrB*), a regulatory protein (*crrA*), and a modulator (*crrC*) that regulates the *pbgP* operon (Fig. 2) (16, 22). An increase in *crrC* expression, mediated by *crrB* mutations, increases PEtN and Ara4N modifications through the *pbgP* operon and the *pmrC* gene, respectively (22, 74). Jayol et al. (74) identified four *crrB* mutations—an F84S mutation within the HAMP domain and N141Y, P151L, and G183V mutations within the histidine kinase A domain, respectively—in four K. pneumoniae isolates, which conferred high-level colistin resistance (74). Each isolate had a colistin MIC value of >128 μg/ml and, when transformed with a plasmid with an intact *crrB* gene, colistin susceptibility was restored (74).

CrrAB regulates *pmrAB* operon through *crrC* expression, and this allows for the activation of both *pmr*C genes and *pbg*P operon through *pmrA* (22, 75). CrrC acts as a connection protein between CrrAB and PmrAB and is regulated by *crrA* (75). Cheng et al. showed that *crrB* mutations also result in an increased expression of H239_3064, a putative efflux pump, resulting in a reduced polymyxin susceptibility (Table 2) (75).

## PhoPQ TWO-COMPONENT REGULATORY SYSTEM

The PhoPQ system encodes a regulatory protein (PhoP) and a membrane-bound sensor kinase (PhoQ); LPS modification is mediated through PhoQ activation of PhoP via phosphorylation (71). Activated PhoP either binds directly to *pbgP* operon or indirectly by binding to PmrD, a connector protein of PhoPQ and the PmrAB system, which protects PmrA from dephosphorylation by PmrB kinase (Fig. 2) (21, 74). The PmrD protein is, however, not found in all *Enterobacteriaceae* species, being mainly found in E. coli, S. enterica, and K. pneumoniae (76). In E. coli, however, PmrD does not connect the two regulatory systems (77).

Similar to PmrB, the cytoplasmic membrane-bound kinase, PhoQ, is activated by environmental stimulants such as a low concentration of magnesium (Mg^2+^) and calcium (Ca^2+^) (69). Jayol et al. showed that an *phoP* mutation, Asp191Tyr, which caused significant modification to the secondary structure of the protein interrupting the α-helix, resulted in elevated colistin resistance (MIC of 12 μg/ml). A *phoP* mutation upregulated *phoP*, *phoQ*, *pmrD*, and *pmrK* (69). Cain et al. demonstrated that a single mutation (K46Q) in the *phoQ* phosphate domain resulted in the loss-of-function of PhoQ, resulting in colistin resistance during serial passage of K. pneumoniae (16). Site-directed mutagenesis in *phoQ* (Leu26Pro) performed by Cheng et al. in K. pneumoniae resulted in elevated colistin resistance with a 32-fold increase in the MIC (Table 2) (68).

Huang et al. (78) investigated the polymyxin resistance mechanisms of heterogeneously resistant E. cloacae using Tn*5* mutagenesis. These authors found that mutations within the DedA protein may mediate heteroresistance to polymyxin through the PhoPQ system (78). The DedA protein is part of a superfamily of membrane proteins and is proposed to be a substrate of the protein-motive-force-dependent drug efflux (78, 79). Though its role in the PhoPQ system was not investigated, Huang et al. found that Tn*5* insertion mutations within the *dedA* gene resulted in E. cloacae susceptibility to polymyxin (MIC of 1 mg/liter). The complementation of *dedA_Ecl_* mutants with plasmids carrying *phoP-phoQ* or wild-type *dedA_Ecl_* with its natural promoters restored the heteroresistance phenotype (MIC of 256 mg/liter) (78).

The polymyxin heteroresistance seen in E. cloacae was, however, mediated by a new small transmembrane protein-encoding gene, *ecr*, which was hypothesized to activate the *pbgP* operon via the PhoPQ system (78). The transformation of Enterobacter mori strain A6008 with *ecr* on a pCR-BluntII-TOPO vector conferred high-level resistance to colistin (MIC 256 mg/liter) and resulted in significant changes in the expression profile of the *pbgP* operon and PhoPQ system (78). Ecr is suspected to act on the PhoPQ system, activating the *pbgP* operon and increasing LPS modification. The introduction of *ecr* into A6008 further resulted in the upregulation of *tolC* and *dedA* expression. Ecr was found to be widely spread in the *Enterobacter* genus, and thus its role in mediating resistance should further be investigated (78).

The *mgrB* gene encodes a small transmembrane lipoprotein that is responsible for the negative regulation of the PhoPQ system by inhibiting the kinase activity of PhoQ (73, 80). Multiple studies have shown that the deletion or inactivation of *mgrB* leads to the upregulation of the *phoPQ* operon, resulting in enhanced LPS modification in K. pneumoniae (Fig. 2) (68, 81, 82). The *mgrB* gene has been reported to be inactivated through various mutations such as deletion, nonsense, missense, and insertional mutations (83–85). Formosa et al. (86) investigated the difference in the surface properties of the extracellular capsule using atomic force microscopy in K. pneumoniae isolates with or without polymyxin. The capsule of the K. pneumoniae isolates with an inactivated *mgrB* gene was tightly bound to the bacterial cell wall and, when exposed to polymyxin, the capsule became harder with increasing concentration. In contrast, polymyxin was able to remove the capsule from K. pneumoniae isolates with an intact *mgrB* gene, resulting in lysis (86). This study demonstrated that inactivation of the *mgrB* gene directly affects the organization of capsules during polymyxin exposure (86).

## MOBILIZED POLYMYXIN RESISTANCE (*mcr*) GENES

The second type of LPS modification seen to achieve polymyxin resistance is the transfer of phosphoethanolamine (PEtN) mediated by PmrC and mobilized colistin resistance (*mcr*-type) genes (21, 82). *mcr*-type genes are plasmid-mediated (some have also been detected on chromosomes) genes that encode enzymes that modify lipid A through the addition of PEtN (23, 87). This phenotype was observed in *mcr-*positive E. coli isolates using mass spectrometry with a PEtN-modified lipid A peak at *m/z* 1,919 instead of *m/z* 1,796 in *mcr-*negative E. coli isolates (87–89). These genes are responsible for the horizontal transfer of polymyxin resistance in *Enterobacteriaceae* through mobile genetic elements (MGEs) (23, 90). To date, there are 10 *mcr* genes that have been identified, with *mcr-1* genes being the most prevalent and predominantly found in E. coli (91, 92). The transformation of E. coli ST7314 isolates with an *mcr-1-*bearing plasmid resulted in a 32-fold increase in colistin MIC (85, 93). The acquisition of *mcr-*type genes, therefore, has a significant clinical impact by conferring high-level resistance to colistin (87). Sato et al. (18) showed that colistin-resistant E. coli had elevated *eptA* and *arnT* expression levels compared to colistin-susceptible E. coli ST131 isolates and exhibited PEtN modifications.

The decrease in polymyxin susceptibility is seen across most *mcr* genes, including *mcr-7.1* isolated from K. pneumoniae in chickens (94), *mcr-5.1* isolated from E. coli in retail chicken rice (95), and *mcr-5* isolated from pigs in E. coli (96). *mcr-4.3*, identified in a clinical E. cloacae isolate in China, was found to not confer polymyxin resistance (97). Chavda et al. (97) compared *mcr-4.3* to *mcr-4* and identified two amino acid substitutions in *mcr-4.3* that significantly altered the function of *mcr-4*, resulting in no modifications to lipid A. *mcr-9*, however, remains the most identified variant after *mcr-1* and is common in several *Enterobacteriales* species, although it is particularly common in Enterobacter hormaechei and other *Enterobacter* sp. (205).

*mcr*-type genes may sometimes be polymyxin induced; Kieffer et al. (98) showed that *mcr-9* mRNA expression was induced by colistin, where an increase in colistin concentration increased the number of *mcr-9* transcripts (98). This feature, however, was only seen with *mcr-9* genes and not with *mcr-1* and was reported to be regulated by the two-component system located downstream of the *mcr-9* gene (98). In *Salmonella* Paratyphi B, Borowiak et al. (99) showed that an increase in plasmid copy number resulted in a higher degree of colistin resistance. Sun et al. (93) showed that one plasmid copy number results in PEtN modification and, subsequently, a reduced polymyxin susceptibility in E. coli isolates; moreover, Zhang et al. (206) demonstrated that the plasmid types hosting the *mcr* gene also affects *mcr* expression and polymyxin resistance.

Kieffer et al. (98) and Cha et al. (92) explored the genomic context of *mcr-9* genes in an E. coli 68A strain and a Salmonella enterica isolate and found that the inducible expression and transferability of *mcr-9* genes was due to the QseC-QseB two-component system. In the E. coli isolate the *mcr-9* gene was located on a IncH2 plasmid, and in S. enterica it was located on an IncX1 plasmid. On both plasmids, the *mcr-9* gene was located between two insertion sequences, and *qseC* and *qseB* genes were located downstream of the *mcr-9* gene (92, 98).

Though a high level of resistance is observed in most *mcr-*positive isolates, Zhang et al. (25) reported that it does not confer the same level of resistance to polymyxin as an inactivated *mgrB* gene. Zhang et al. (25) further reported that the transformation of inactivated *mgrB* K. pneumoniae isolates with an *mcr-*harboring plasmid does not result in a synergistic activity i.e., no change in polymyxin MIC value. Sato et al. (18) and Kieffer et al. (98) both showed that *mcr*-type genes do not affect PmrAB genes; thus, only PEtN modifications are observed in *mcr-*positive isolates (Fig. 2).

## POLYMYXIN RESISTANCE INDUCTION

In polymyxin inducing environments, *mcr-*negative isolates acquire resistance through mutations that increase the expression of PhoPQ and PmrAB regulatory systems (71). Mutations within the *phoQ* and insertions in *mgrB* have been identified in K. pneumoniae isolates, resulting in the upregulation of *phoQ*, *pmrD*, and *pbgP* (71). A K. pneumoniae isolate was identified with *crrB* mutations resulting in increased *pmrB* expression (71). A serial passage performed by Cain et al. (16) reported a change in membrane permeability, contributing to polymyxin resistance. The expression of several efflux pumps—BN373_11321 (RND-family), BN373_15271 (MacA), and BN373_26531 and BN373_36071 (RND-family)—was increased under polymyxin selection (16). These efflux pumps have been previously reported to act as multidrug transporters (16). The outer membrane porins OmpA and OmpC that allow for active antibiotic uptake were significantly decreased in K. pneumoniae isolates (16). Although these mechanisms were not actively investigated, the change in membrane permeability may result in the reduction of polymyxin susceptibility (Table 2).

## CONCLUSION

The misuse of antibiotics creates a selection pressure, resulting in mutations or transmission of resistance genes mediating antibiotic resistance. This is mainly seen in β-lactamase- and *mcr*-negative *Enterobacteriaceae* isolates that mutate and acquire high-level resistance in antibiotic-inducing environments through target modifications, overexpression of efflux pumps, and loss of major porin groups. This emphasizes the need for the correct usage of carbapenem and polymyxin antibiotics during therapy to ensure therapeutic success instead of the production of resistant clinical isolates. This review provides an in-depth molecular characterization of current and emerging resistance mechanisms that mediate carbapenem and polymyxin resistance. The mechanisms, however, of RamA, an efflux pump regulator, and *micC* and *micF*, which are small RNAs, in downregulating the major porin groups is unknown. Thus, further research into factors influencing this phenotype through these negative regulators is required. The role of *micC* and *micF* also shows the importance of sRNA and siRNA in gene regulation and antimicrobial resistance. Hence, studies investigating the global role of these small regulatory RNAs in microbial resistance is needed.

Within *Enterobacterales*, members of the tribe *Proteeae* and *Serratia* sp. are known to be intrinsically resistant to polymyxin. Mechanisms mediating resistance to these species were not discussed here since they were not found in the included articles. Future studies might interrogate transcriptional, coding, and noncoding genetic elements mediating intrinsic resistance in these clinically important species. Finally, studies demonstrating the transcriptional effects of noncoding and coding genetic elements in OXA-48 carbapenemase-mediated carbapenem resistance were not included in this review because they did not meet the inclusion criteria. Hence, further transcriptional and mutagenesis studies are required to confirm the effects of noncoding and coding genetic elements on the transcriptional levels and subsequent phenotypic resistance levels of OXA-48-type carbapenemases in *Enterobacterales*.
