# Supplementary figures and images for: Emerging Transcriptional and Genomic Mechanisms Mediating Carbapenem and Polymyxin Resistance in Enterobacteriaceae: a Systematic Review of Current Reports
Source: mSystems. 2020 Dec 15;5(6):e00783-20. doi: 10.1128/mSystems.00783-20 (PMC7771540; doi:10.1128/mSystems.00783-20)

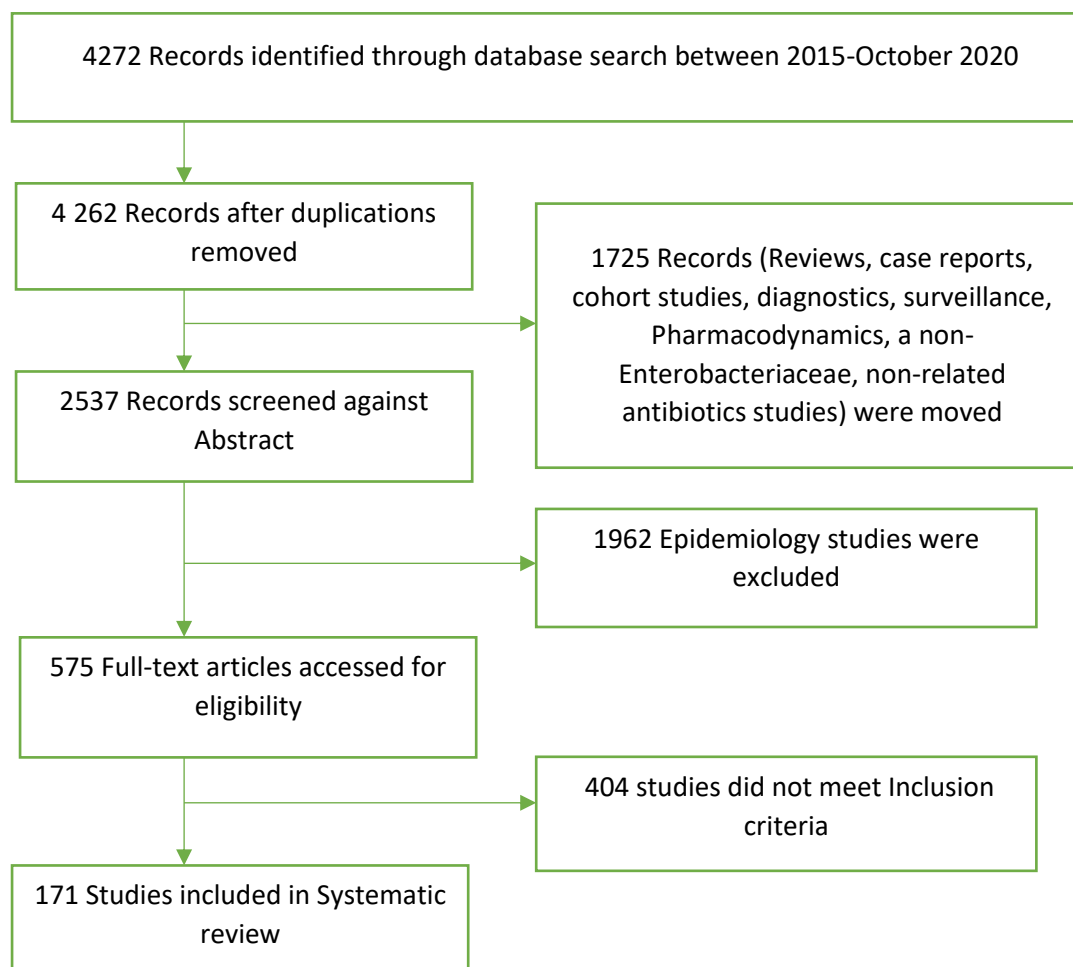

Supplement: FIG S1 [file mSystems.00783-20-sf001.pdf]
